# 

**Published:** 1889-08

**Authors:** 


VOL. XIX. AUGUST, 1889. NO. 8

### Southern Medical Record.

EDITORS.

*T. S. POWELL. M. D.* *R. C. WORD. M. D.*

COLLABORATORS:

J. McF. Gaston, M. D.,
Julian J. Chisolm, M. D.,
W. P. Nicolson, M. D.,
E. Van Goidtsnoven, M. D.,
P. R. Cortelyou, M. D.,
Lindsay Johnson, M. D.,
G. G. Roy. M. D.,
A. G. Hobbs, M. D.,
W. S. Elkin, M. D.,
W. A. Crow. M. D.,
Jno. Thad Johnson, M. D.,
K. P. Moore, M. D.

**Terms:—$2.00 per annum, in advance; four copies $6.00; single copies 20 cents.**

**Address R. C. Word, M. D. Managing Editor, Atlanta, Ga.**

Published and entered as Second Class Matter at Atlanta, Ga.

